# Role and clinical implication of autophagy in COVID-19

**DOI:** 10.1186/s12985-023-02069-0

**Published:** 2023-06-16

**Authors:** Tianjiao Shan, Lan-ya Li, Jin-Ming Yang, Yan Cheng

**Affiliations:** 1 Department of Pharmacy, The Second Xiangya Hospital, Central South University, Changsha, 410011 China; 2 Hunan Provincial Engineering Research Centre of Translational Medicine and Innovative Drug, Changsha, 410011 China; 3 Department of Toxicology and Cancer Biology, Department of Pharmacology, and Markey Cancer Center, University of Kentucky, Lexington, KY 40536 USA

**Keywords:** Autophagy, Clinical treatments, Coronavirus, COVID-19, Infectious diseases, SARS-CoV-2, Vaccines

## Abstract

The ongoing coronavirus disease 2019 (COVID-19) pandemic constitutes a serious public health concern worldwide. Currently, more than 6 million deaths have occurred despite drastic containment measures, and this number is still increasing. Currently, no standard therapies for COVID-19 are available, which necessitates identifying effective preventive and therapeutic agents against COVID-19. However, developing new drugs and vaccines is a time-consuming process, and therefore, repurposing the existing drugs or redeveloping related targets seems to be the best strategy to develop effective therapeutics against COVID-19. Autophagy, a multistep lysosomal degradation pathway contributing to nutrient recycling and metabolic adaptation, is involved in the initiation and progression of numerous diseases as a part of an immune response. The key role of autophagy in antiviral immunity has been extensively studied. Moreover, autophagy can directly eliminate intracellular microorganisms by selective autophagy, that is, “xenophagy.” However, viruses have acquired diverse strategies to exploit autophagy for their infection and replication. This review aims to trigger the interest in the field of autophagy as an antiviral target for viral pathogens (with an emphasis on COVID-19). We base this hypothesis on summarizing the classification and structure of coronaviruses as well as the process of SARS-CoV-2 infection and replication; providing the common understanding of autophagy; reviewing interactions between the mechanisms of viral entry/replication and the autophagy pathways; and discussing the current state of clinical trials of autophagy-modifying drugs in the treatment of SARS-CoV-2 infection. We anticipate that this review will contribute to the rapid development of therapeutics and vaccines against COVID-19.

## Introduction

Coronavirus disease-2019 (COVID-19) is a newly emerged contagious disease caused by severe acute respiratory syndrome coronavirus 2 (SARS-CoV-2), which has caused millions of deaths and hospitalizations worldwide. The characteristics of SARS-CoV-2 infection in humans are lung infection accompanied by various clinical manifestations, including fever, cough, fatigue, dyspnea, and headache, varying from asymptomatic or mild respiratory symptoms to severe pneumonia [1]. Although the symptoms of COVID-19 are usually mild, sepsis, acute respiratory distress syndrome (ARDS), and cytokine storm complications associated with SARS-CoV-2 infection can be fatal [2]. Since late 2019, SARS-CoV-2 spread rapidly worldwide and has been presenting a global public health challenge, prompting the World Health Organization (WHO) to declare it as a pandemic [3, 4]. By February 18, 2022, more than 418 million COVID-19 cases were officially diagnosed, resulting in over 5.8 million deaths (WHO 2021). As of February 20, 2022, at least nine different vaccines across seven platforms have been rolled out in different countries. Given the severity of this pandemic, the research and development of COVID-19 vaccines have been greatly shortened compared with that of traditional vaccines. SARS-CoV-2 is an RNA virus with a high mutation rate, thus challenging the effectiveness of existing vaccines. As reported by Chen et al., monoclonal and serum-derived polyclonal antibodies showed reduced inhibitory activity against SARS-CoV-2 variants containing E484K spike mutation [5]. Thus, a better understanding of COVID-19 pathogenesis is essential to treat the infection.

Macroautophagy (known as autophagy) is a highly conserved lysosome-dependent degradation process in which intracellular components are encapsulated in a double-membrane autophagosome and then transported to the lysosome to remove unwanted or harmful substances and recycle useful materials [6]. Autophagy can eliminate long-lived proteins and damaged organelles when it occurs at basal conditions for maintaining cellular homeostasis [7]. Autophagy is an adaptive process occurring in response to various environmental stresses including starvation, oxidative stress, and pathogen infections [8]. Autophagy upregulation provides cells with nutrition for energy supply and biosynthesis and removes potentially cytotoxic substances. Autophagy has a wide range of physiological and pathological implications in many human diseases including cancer, neurodegenerative diseases, and infections. Recently, mounting evidence has shown that autophagy is a powerful means for host cells to evade viral infection. Autophagy is activated by the innate immune system to counteract viral infection, degrading invading viruses and facilitating antigen processing and adaptive immune responses [9]. However, some RNA viruses, such as hepatitis C, Zika, SARS-CoV, and Middle East respiratory syndrome coronavirus (MERS-CoV), can use autophagosomes for their replication [10–12]. It will be of great significance to discuss how to modulate the autophagy pathway as a potentially beneficial therapeutic strategy to prevent and combat COVID-19. The use of chloroquine (CQ) and hydroxychloroquine (HCQ), two known autophagic inhibitors, for treating SARS-CoV-2-infected patients raised questions about the role of autophagy in COVID-19 pathogenesis [13]. In addition, other autophagy modulators are being actively tested in vitro and in vivo for COVID-19-related treatments. Interestingly, intermittent fasting (IF) has been proposed as a promising preventive approach against SARS-CoV-2 infection because this dietary restriction activates autophagy and triggers priming of the immune response [14].

In this article, we discuss the role of autophagy in the pathogenesis of SARS-CoV-2 infection, the antiviral functions of this important cellular process, and the potential of autophagy as a therapeutic target for COVID-19.

## SARS-CoV-2 infection

### SARS-CoV-2 virus

Coronaviruses (CoVs) are the largest class of viruses in Nidovirales with spike-like projections; hence the name [15]. They are enveloped single-stranded RNA viruses that spread widely among mammals and birds [16]. The disease caused by coronaviruses can be categorized into four genera: α-, β-, γ-, and δ-CoVs; among them, α- and β-CoVs mainly infect mammals, whereas γ- and δ-CoVs mainly infect birds [17]. By December 2019, six CoVs including HCoV-229E, HCoV-OC43, SARS-CoV, HCoV-NL63, HCoV-HKU1, and MERS-CoV were identified as the viruses causing infection in humans. SARS-CoV and MERS-CoV caused severe pneumonia in humans and were responsible for the epidemic outbreaks in 2002 and 2012, respectively, whereas the rest contributed to mild upper respiratory symptoms, such as common cold, in humans [18, 19]. SARS-CoV-2, similar to SARS-CoV and MERS-CoV, belongs to the β-CoV genus and shows ~ 80% sequence similarity to SARS-CoV and ~ 50% sequence similarity to MERS-CoV according to the phylogenetic analysis [20]. SARS-CoV-2 has 14 open reading frames (ORFs) encoding 27 proteins, and ORF1a and ORF1b located at the 5′ end of the SARS-CoV-2 genome are responsible for encoding 16 nonstructural proteins (NSP 1–16). Four structural proteins, spike glycoprotein (S), envelope (E), membrane (M), and nucleocapsid (N), are encoded by ORFs located at the 3′ end of the genome [21]. The transmembrane protein spike is incorporated into the lipid envelope of CoVs, which recognizes its receptor specifically by a receptor-binding domain, leading to virus entry into host cells [20]. The S protein contains two functional subunits: S1 is responsible for interacting with its receptors, and S2 is responsible for the fusion of the viral envelope with host cell membrane. A polybasic furin-type cleavage site exists at the S1–S2 junction in the S protein of SARS-CoV-2 but not in that of SARS-CoV. Following binding with its receptor, the S protein is cleaved at the special site by transmembrane protease serine 2 (TMPRSS2) or endolysosomal cathepsin L of target cells, facilitating the proteolytic activation of the S protein and virus expansion, which may explain the enhanced spreading and pathogenicity of SARS-CoV-2 [22, 23]. The N protein of CoVs is an RNA-binding protein and consists of three highly conserved parts, namely the N-terminal RNA-binding domain for RNA binding, the C-terminal dimer domain for oligomerization, and a Ser/Arg-rich linker for primary phosphorylation. The N protein plays a key role in viral RNA transcription and replication [24]. The viral M protein exists in the virion as a dimer and has two different conformations involved in the budding process, namely envelope formation and virus particle assembly [25]. The E protein, a glycoprotein, is an 8–12 kDa transmembrane protein and possesses ion channel activity involved in the assembly and release of virions [26].

### The process of SARS-CoV-2 infection and replication

**Fig. 1:**
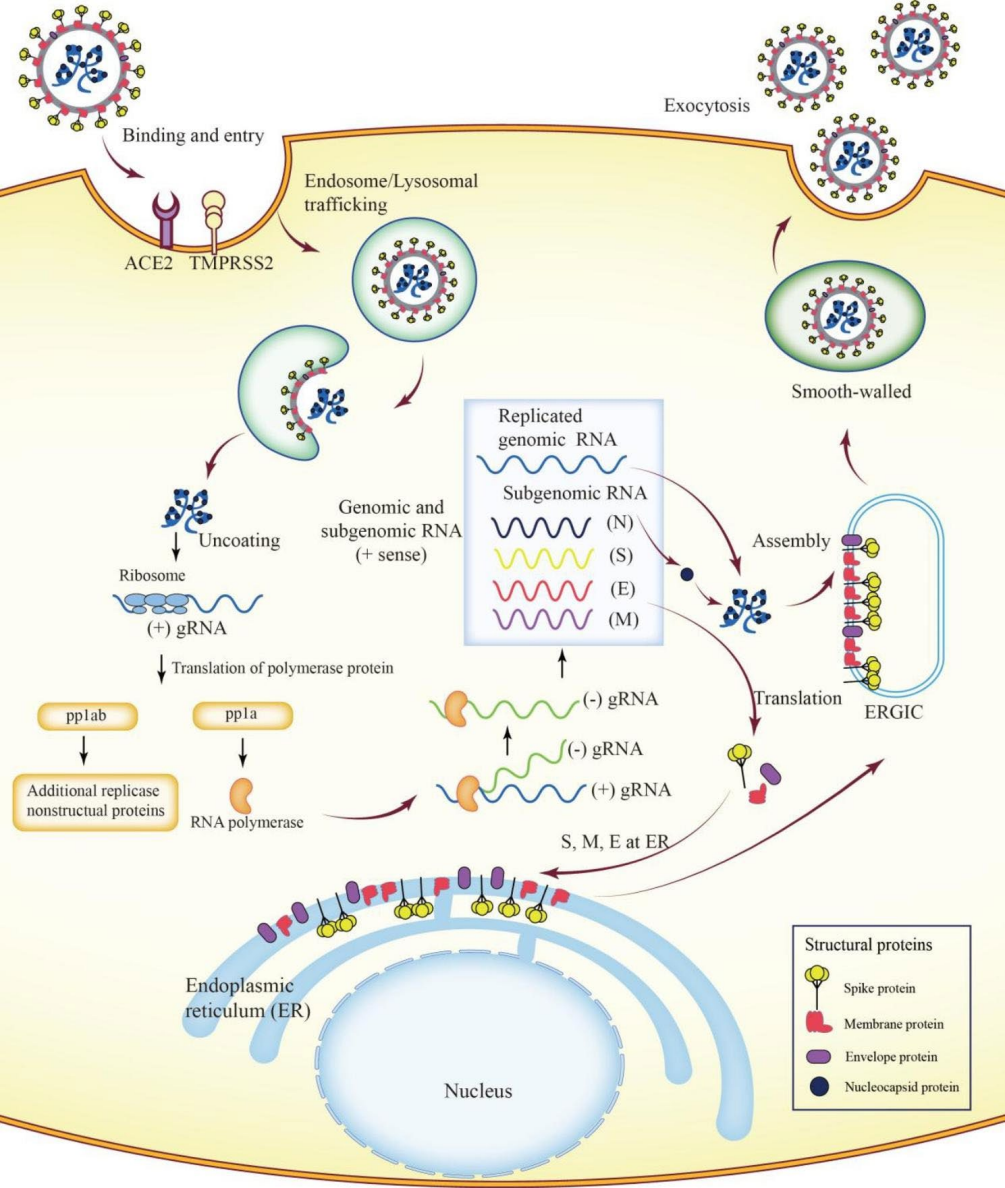
Replication cycle and infection process of SARS-CoV-2 Virus entry into host cells is the first step of severe acute respiratory syndrome CoV 2 (SARS-CoV-2) infection. The entry of SARS-CoV-2 is mediated by the combination of S protein with the host cell surface receptor angiotensin-converting enzyme 2 (ACE2) with the aid of the enzyme transmembrane protease serine 2 (TMPRSS2). Subsequently, the virus entering host cells fuses with the lysosomal membrane, and the viral nucleocapsid is uncoated to be exposed in the cytoplasm, which marks the beginning of virus replication. The positive-sense genome RNA, which serves as the first mRNA of infection, replicates into complete virions processed through the host cell nuclear, ER, and ER–Golgi intermediate complex (ERGIC). Full-length genomic RNA (gRNA), also known as the positive-sense genome, is replicated as the template for the synthesis of progeny genomes and a nested set of subgenomic RNA (sgRNA) via a negative-sense intermediate using RNA polymerase (RdRP). The gRNA can be translated into the polyproteins pp1a and pp1ab, which are cleaved to form individual replicate complex NSP. sgRNAs encode viral structural and accessory proteins, among which the membrane-bound structural proteins M, S, and E are inserted into the ER and then into the ERGIC. Finally, mature progeny virions in smooth-walled vesicles are transported to the plasma membrane and released by exocytosis to perform their function Abbreviations: ACE2: angiotensin-converting enzyme 2; E: envelope; ER: endoplasmic reticulum; ERGIC: ER–Golgi intermediate complex; gRNA: genomic RNA; M: membrane; NSP: nonstructural proteins; RdRP: RNA-dependent RNA polymerase; S: spike glycoprotein; SARS-CoV-2: severe acute respiratory syndrome CoV 2; sgRNA: subgenomic RNA; TMPRSS2: transmembrane protease serine 2

SARS-CoV-2 entry into the human body and its replication can be divided into the following steps: adsorption and endocytosis; uncoating; synthesis and assembly; and release (Fig. 1). The interaction between S protein and its host receptors is the first step of SARS-CoV-2 infection. The sequence of the S protein of SARS-CoV‐2 and SARS‐CoV shares 76% identity [27]. Thus, the spike proteins of both can interact with angiotensin-converting enzyme 2 (ACE2) for entering cells, although the binding affinity of SARS‐CoV‐2 toward ACE2 is higher than that of SARS-CoV [28]. ACE2 is a type I membrane protein and is widely expressed in the lung, heart, kidney, intestine, and gut [29, 30]; however, it is highly expressed in lung type II alveolar cells, oral epithelial cells, upper esophagus cells, and bile duct cells, suggesting that organs with high ACE2-expressing cells are at a high risk of SARS‐CoV-2 invasion [30–32]. In addition, other potential entry receptors of SARS‐CoV-2, including CD147, CD209, and CD209L, have been identified [26]. Following binding with ACE2, virus particles are endocytosed by host cells, and viral membranes are fused with host cells, releasing viral genomic RNA into the cytoplasm of infected cells [21]. CoV, similar to other virus families, replicates its RNA and synthesizes viral proteins by hijacking host cells. The uncoated positive-strand RNAs containing ORF1a and ORF1b are immediately used as a template and translated into two polyproteins (pp1a and pp1ab). Subsequently, 16 NSPs are produced when these polyproteins are cleaved by two cysteine proteases located within NSP3 (papain-like protease; PL^pro^) and NSP5 (chymotrypsin-like protease, M^pro^). The NSP12 gene encodes RNA-dependent RNA polymerase (RdRP). These multifunctional NSPs promote viral RNA replication in double-membrane vesicles (DMVs) derived from the endoplasmic reticulum (ER). Viral particles are assembled in the ER and Golgi apparatus [20, 33].

### Current treatment options for COVID-19

The most common symptoms of SARS-CoV-2 infection are fever, fatigue, cough, and dyspnea. However, some patients experience severe pneumonia, which progresses to ARDS, lung damage, multi-organ failure, and even death [33–35]. The pathogenesis of severe COVID-19 relies on the host–virus interaction, leading to massive inflammatory cell infiltration and proinflammatory cytokine release (termed as cytokine storm) [36]. Based on the pathogenesis and clinical symptoms of COVID-19, a growing number of Food and Drug Administration (FDA)-approved drugs and biologics are being tested in clinical trials, which include antivirals, S protein cleavage inhibitors, and selective and nonselective immunosuppressors (Table 1). Remdesivir, an antiviral drug belonging to the class of nucleotide analogs, has broad-spectrum activity against several members of the virus family. It was previously used to treat infections caused by CoVs including SARS-CoV and MERS-CoV and is currently being used for treating moderate and severe COVID-19. Other antiviral drugs such as lopinavir, arbidol, favipiravir, and camostat (TMPRSS2 inhibitor) are being tested for their efficacy against COVID-19 [33, 35]. Dexamethasone can regulate inflammation-mediated lung injury, reducing the progression of respiratory failure and death. Dexamethasone is also used clinically to treat SARS-CoV-2 infection. Interferon beta-1b in combination with lopinavir–ritonavir and ribavirin has shown better efficacy than lopinavir–ritonavir alone in patients with mild-to-moderate COVID-19 [33, 37]. Vaccines shall be the most effective approach for preventing infectious diseases as they can reduce morbidity and mortality. Currently, nine COVID-19 vaccines have been approved for use. Until February 20, 2022, a total of 10.3 trillion doses of all kinds of vaccines have been administered worldwide. Although seeing many vaccines going into development is encouraging for the public, research on improving the protective efficacy against virus-variant vaccines is still ongoing. Furthermore, the WHO, accompanied by national authorities, is developing and implementing standards to guarantee COVID-19 vaccine safety. No vaccine is 100% effective, and the risk of reinfection and retransmission in vaccinated people remains uncertain. Overall, despite the widespread availability of vaccines, ensuring public health and social measure practices is important.

**Table 1:** The current available drugs targeting COVID-19 infection

Drug	Mechanism	Type of Study	Refs
IFN-β1a (SNG001)	Promote the innate immune response of human lung.	Clinical trial/ Phase II	[116]
Tocilizumab	A recombinant human IL-6 monoclonal antibody that specifically binds to IL-6 receptors and inhibits IL-6-mediated signal transduction.	Clinical trial	[117]
IFN-α2b	Interfere with virus infection and replication.	Laboratory Tests	[118]
Fluvoxamine	A strong S1R agonist to reduce inflammatory response through the S1R-IRE1 pathway.	Clinical trial	[119]
Combination of IFN-β1b, lopinavir-ritonavir, and ribavirin	A multiple antiviral drugs combination to improve the viral load profile.	Clinical trial/ Phase II	[120]
Vitamin C	Block several key components of cytokine storms.	Clinical trial	[121]
Hydroxychloroquine	Increase endosome pH to prevent virus entry and interfere with the glycosylation of ACE2.	Clinical trial	[48]
Remdesivir	A prodrug of an adenosine analogue having antiviral activity against various RNA viruses.	Clinical trial/ Phase III	[122]
Dexamethasone	An anti-inflammatory drug and immunosuppressants.	Clinical trial	[123]
Molnupiravir	A prodrug of the ribonucleoside analogue β-D-N4-hydroxycytidine having broad-spectrum antiviral activity	Clinical trial/ Phase II	[124]

**Abbreviations**: ACE2: angiotensin-converting–enzyme 2; COVID-19: coronavirus disease 2019; IFN: Interferon; IL: Interleukin; IRE1: inositol-requiring enzyme 1α; S1R: σ-1 recept

## Autophagy, an essential process to maintain cellular homeostasis and functions

**Fig. 2:**
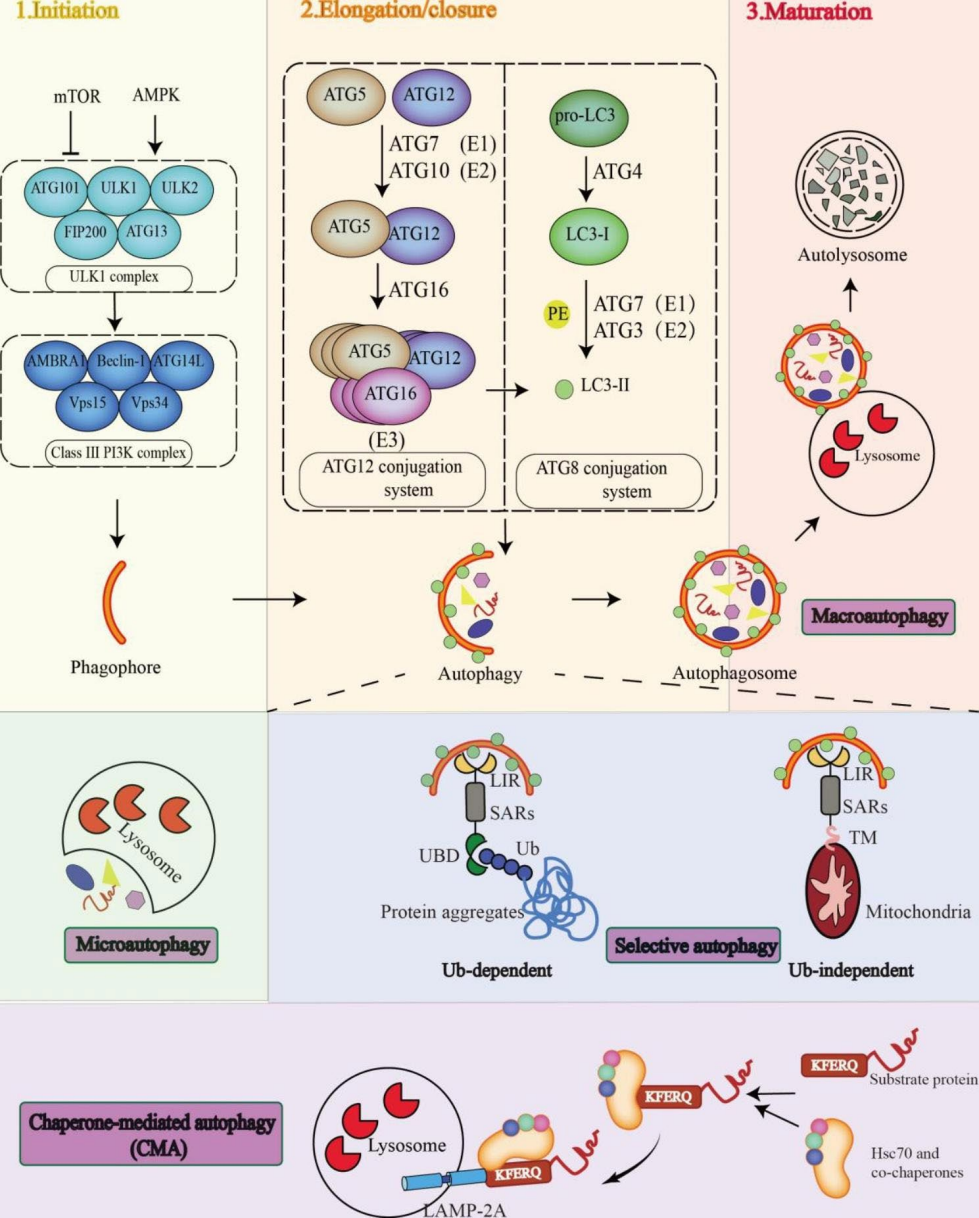
The types and processes of autophagy Autophagy is mainly divided into three classical types, namely macroautophagy, microautophagy, and CMA. ①Macroautophagy: The procedures of macroautophagy can be characterized by initiation, elongation/closure, and maturation according to the obvious morphological changes in vesicular compartments. PGs are formed from intracellular membranes regulated by ULK1 complex and class III PI3K complex, and subsequently, they are transformed into AVs enriching the LC3-II regulated by the conserved autophagy genes. Macroautophagy was considered to be a process that disposes cytoplasmic components into AVs randomly and nonselectively, whereas the selective function of AVs has been garnering increasing attention recently. In selective autophagy, cargo is linked to AVs by a physical combination of SARs and LC3-II through the LIR motif and recognized in an Ub-dependent or Ub-independent manner. Finally, the autophagosomes fuse with the lysosomes to form the autolysosomes where the cargoes are degraded and the degradative products released back into the cytosol are reused. ②Microautophagy: Cargoes, including proteins and organelles, are sequestered and degraded in bulk through direct invagination into the lysosome. ③CMA: The cytosolic substrates containing the pentapeptide KFERQ motif are transferred to the lysosome for internalization and degradation when they are recognized in an HSPA8/HSC70 chaperone-dependent manner and thus bind with the LAMP-2 A multimeric complex on the lysosomal membrane Abbreviations: AMBRA1: autophagy and beclin 1 regulator 1; ATG: autophagy-related; AVs: autophagic vesicles; CMA: chaperone-mediated autophagy; FIP2000: focal adhesion kinase family interacting protein of 200kDa; LAMP-2A: lysosomal-associated membrane protein 2A; LC3-II: LC3 lipid; LIR: LC3/GABARAP-interacting region; mTOR, mechanistic target of rapamycin; NSP, nonstructural protein; PE: phosphatidylethanolamine; PGs: phagephore; PIK3C3/VPS34: phosphatidylinositol 3-kinase catalytic subunit type 3; PI3K: phosphoinositide 3-kinase; SARs: selective autophagy receptors; Ub: ubiquitin; UBD: ubiquitin binding domain; ULK, unc-51 like autophagy activating kinase

Autophagy is a fundamental self-digesting cellular event that transports various intracellular constituents to the lysosome for degradation and recycling (both types and processes of autophagy were described in Fig. 2). It is implicated in many pathophysiological processes including development, differentiation, survival, and homeostasis via eliminating unnecessary and potentially harmful substances. Autophagy was initially considered an adaptive response to nutrition deprivation, providing molecules needed for anabolism. Originally, it was considered a nonselective means to destroy bulk cytoplasmic components [38]. However, with a better and deeper understanding of autophagy, scholars have asserted that it is not a random process of phagocytizing the cytoplasm indiscriminately but involves selective targeting of substrates corresponding to a specific stimulus, thus maintaining cellular homeostasis and supporting cell survival [8, 39]. Multiple types of selective autophagy have been reported, including mitophagy (degradation of mitochondria), pexophagy (selective removal of peroxisomes), aggrephagy (selective degradation of protein aggregates), reticulophagy (selective degradation of the ER), lysophagy (selective degradation of damaged lysosomes), and xenophagy (selective removal of microorganisms) [40].

### Autophagy machinery

In eukaryotic cells, autophagy is tightly regulated by autophagy-related (ATG) proteins and protein complexes, as well as a multistep and complex process, requiring the coordination of several molecules and rearrangement of intracellular membranes for delivering cellular materials to lysosomes for breakdown. Autophagy activation involves initiation, elongation/closure, and maturation (Fig. 2). The initial step is the recruitment of proteins to the phagophore assembly site (PAS) and expansion of the membrane to form a double­membrane, cup-shaped phagophore (PG) (initiation step). Subsequently, an autophagosome is formed from the PG, which is a sealed vesicle that can engulf cargo (elongation step). The formation of the autophagosome, a unique, double-membrane organelle, is a marker of autophagy. Finally, autophagosomes fuse with lysosomes, and cellular substances are degraded by lysosomal enzymes and reused by anabolic pathways (maturation step) [41–43].

Autophagy activation is tightly regulated by multiple signaling pathways, as well as dynamic membrane complexes containing ATG genes and proteins. Autophagy can be triggered by multiple stressors such as ER stress, nutritional restriction, and pathogen infection and is regulated by signals such as nutrition (mTOR), energy (AMPK), and stress (HIFs), which can turn on or off autophagy pathways [44]. The ULK1 complex (ULK1-ATG13-FIP200-ATG101) is a signal initiation complex of autophagy. Under stress or nutritional deficiency, mTORC1 is inactivated and releases ULK1, which activates the autophagy signal [45]. Upon induction, the ULK1 complex phosphorylates and activates the beclin (BECN1)-VPS34 complex, including BECN1-VPS34-VPS15-ATG14 [46]. The lipid kinase VPS34 produces a phosphatidylinositol 3-phosphate (Ptdins3P)-rich region on the surface of the autophagy donor membrane, including the ER, Golgi body, and ER–mitochondrial contact point, and recruits the Ptdins3P-binding proteins WIPI1–WIPI4 to PAS [47]. Two ubiquitin-like conjugation systems are responsible for autophagosome extension and expansion. The ubiquitin-like conjugate of ATG5 and ATG12 is activated by ATG7 and ATG10. ATG16L1 forms a complex with ATG5-ATG12 in the noncovalent form, which is related to the expanding PG membrane [48, 49]. The second ubiquitin-like conjugate is LC3-phosphatidylethanolamine (PE) (also known as LC3-II), which is mediated by ATG7, ATG3, and Atg16L1 complexes when the C-terminal arginine of LC3 is cleaved by the cysteine protease ATG4B and processed into soluble LC3-I [50, 51]. This lipid-binding form of LC3 promotes autophagy and can be used as an autophagic marker .

### Selective autophagy and selective autophagy receptors

The key components involved in selective autophagy include the core autophagy machinery and selective autophagy receptors (SARs) that specifically recognize the cargo and deliver substrates to Atg8-family proteins on the PG to degrade the cargo [52]. Generally, the LC3/GABARAP-interacting region (LIR) motif of SARs is essential for their binding with Atg8 homologs, ensuring selective sequestration of cargo [53]. Ubiquitination plays a key role in the recognition and degradation of a substrate protein by selective autophagy [54]. SQSTM1/p62 (sequestosome 1) is the first identified SAR [55]. It possesses the LIR motif and ubiquitin-binding domains, functioning as a bridge between the ATG8-family proteins and the ubiquitinated substrates [56]. Currently, more than 30 cargo receptors have been identified in mammals, such as SQSTM11, NBR1, OPTN (Optineurin), NDP52, and TAX1BP1. These receptors can recognize and bind to specific ubiquitin-binding proteins and then deliver the autophagic cargo to the site of autophagosomal engulfment containing LC3. SARs comprise soluble receptors, such as the abovementioned SARs and membrane-associated receptors [57]. Multiple proteins including BNIP3L, FUNDC1, BNIP3, and FKBP8 localize in the mitochondrial membrane and act as mitophagy receptors to directly recognize LC3 by its LIR and promote the breakdown of the dysfunctional mitochondria [58–60]. Autophagic receptors are not limited to proteins with an LIR motif or those that bind to LC3. Recently, a novel SAR-Atg8 binding method was identified: the receptor Arabidopsis RPN10 involved in recruiting inactive 26S proteasomes could bind to the ubiquitin-interacting motif (UIM)-docking site of Atg8 through its unrelated UIM [61]. Studies on IFN-γ-induced selective autophagy have identified novel autophagic receptors—the tripartite motif (TRIM) proteins TRIM20 and TRIM21—which could bind to specific cargo directly via their PRY/SPRY domain and serve as a platform to recruit activated autophagic components, leading to cognate target degradation [62]. Most SARs are regarded as proteins, but evidence suggests that cardiolipin and ceramide act as the receptor in mitophagy [40]. OPTN and NDP52 are ubiquitin-binding autophagy receptors that also function in promoting the recruitment of the ULK1/2-Atg13-FIP200-Atg101 complex during PINK1/Parkin mitophagy [53]. The cargo receptor NDP52 involved in xenophagy functions in recruiting the upstream autophagy machinery to bacteria initially by forming a trimeric complex with FIP200 and SINTBAD/NAP1, resulting in PG formation in situ and subsequent anti-bacterial autophagy steps [63].

## Autophagy in CoV infection

**Fig. 3:**
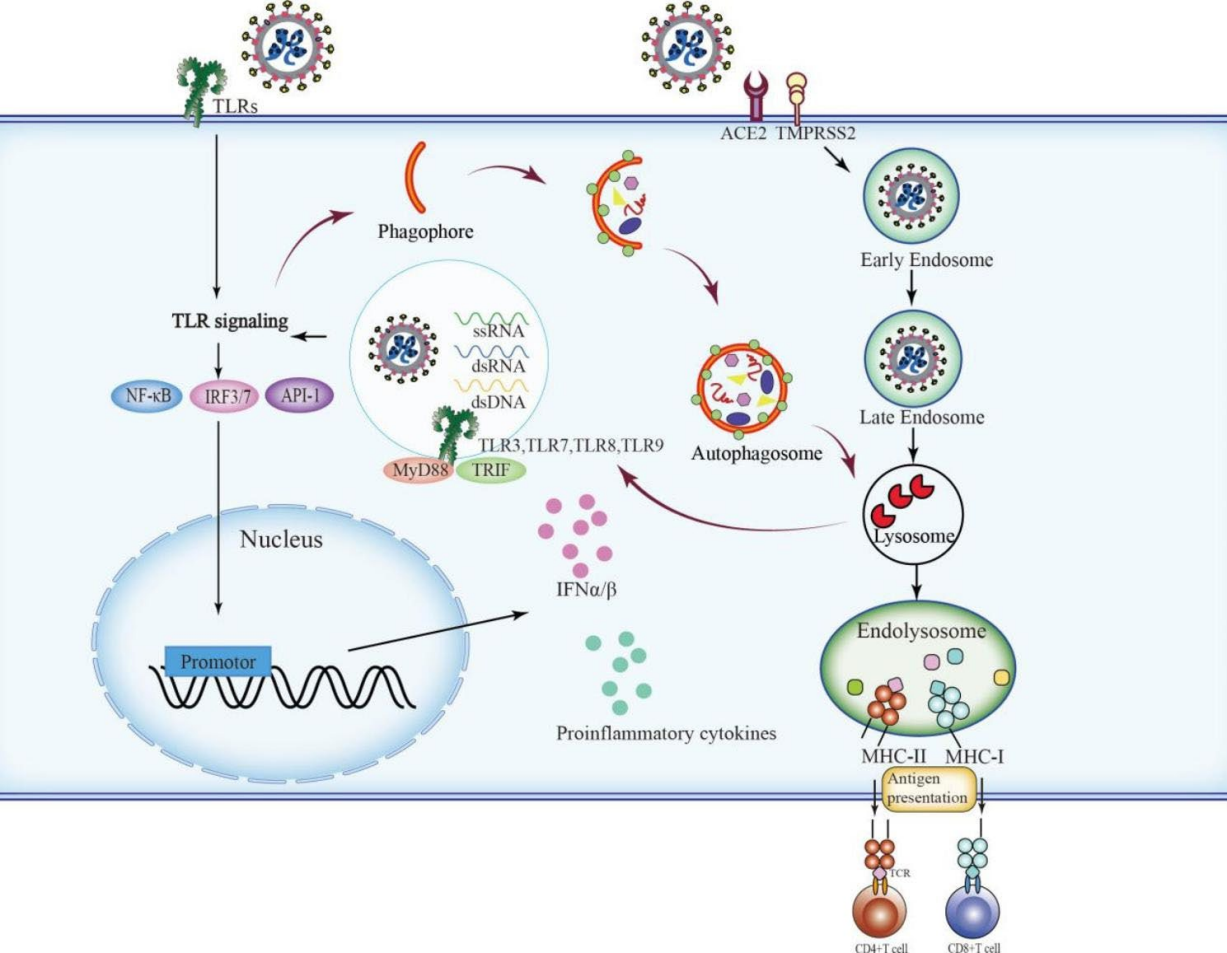
Autophagy in antiviral immune responses The innate immune system is the first line of defense in multicellular organisms that initiates proinflammatory responses to protect against host tissue damage and microbial invasion such as virus infection. Along with the enhancement of this innate response, autophagy is activated to trap specific pathogens and transport them into autophagosomes for degradation. During viral infection, TLRs of the PRRs family are the primary proteins responsible for initiating innate immune responses and inducing autophagy in mammals. TLR signaling increases the interaction of MyD88 or TRIF with BECN1 and promotes the dissociation of BECN1 from Bcl-2 to promote autophagy. TLR-induced autophagy belongs to selective autophagy (known as xenophagy) that occurs in a ubiquitin-dependent manner. When the virus enters the organism, the viral nucleocapsid is uncoated to be exposed in the cytoplasm by the late endosome or lysosome. TLR signaling pathways are activated by the unique dsRNA, ssRNA, or dsDNA of invading viruses with decapsulated capsid. Interaction between TLRs and MyD88 or TRIF, both of which are responsible for the activation of transcription factors including NF-κB, IRF3/7, and API-1 triggers IFN-I generation and proinflammatory cytokines secretion, which are key immune responses against viruses, and further stimulates factors for innate immune responses. Additionally, autophagy contributes to antigen presentation. In detail, autophagy promotes the upload of the peptides derived from the pathogen into MHC-II molecules for presentation to CD4^+^ T cells, in addition to participating in an alternative pathway of class I presentation, also termed cross-presentation Abbreviations: BECN1: beclin1; dsRNA: double-stranded RNA; MHC-II: major histocompatibility complex class II; MyD88: myeloid differentiation primary response gene 88; NF-κB: nuclear factor kappa B; PRRs: pattern recognition receptors; ssRNA: single-stranded RNA; TLRs: Toll-like receptors; TRIF: TIR domain-containing adaptor molecule 1

Autophagy is an important housekeeping mechanism required to maintain host health that facilitates the clearance of invaded pathogens by the immune system. Notably, autophagy can be rapidly activated in response to stress caused by viral infection. During infection, selective autophagy is initiated (termed xenophagy) to capture specific foreign viral proteins into PG and subsequently package them into autophagosomes, followed by their elimination by lysosomes (Fig. 3).

### Autophagy in antiviral immune responses

The innate immune system, the first line of defense in organisms, initiates proinflammatory responses to protect against host tissue damage and microbial invasion such as viral infection. As a major degradation pathway, autophagy can be activated by the innate immune system to arrest and dispose of foreign viruses. During viral infections, the innate immune system can rapidly detect the presence of viruses by pattern recognition receptors (PRRs), which recognize pathogen-associated molecular patterns (PAMPs) of invading pathogens. Subsequently, the transduction of intracellular signals is initiated to activate the factors involved in regulating the synthesis of inflammatory cytokines, culminating in the stimulation of innate immune responses.

Along with the enhancement of innate immune responses, autophagy is activated to trap specific pathogens and deliver them to autophagosomes for degradation. Several innate immunity-related proteins are involved in the regulation of autophagy. During viral infection, Toll-like receptors (TLRs) of the PRR family are the primary proteins responsible for initiating innate immune responses and inducing autophagy in mammals [64, 65]. After sensing the PAMPs of viruses, TLRs trigger antiviral immune responses by interacting with the adaptor’s myeloid differentiation primary response gene 88 (MyD88) or TIR domain-containing adaptor molecule 1 (TRIF) [66]. Both adaptors are responsible for the activation of nuclear factor-κB, IRF3/7, and API-1, the transcription factors involved in proinflammatory cytokine synthesis and IFN production. TLR signaling can enhance the interaction of MyD88 or TRIF with BECN1 and promote the dissociation of BECN1 from Bcl-2, leading to autophagy [67].

Autophagy induced by TLR signaling is selective (known as xenophagy) and requires a SAR to detect and capture cargo for selective degradation. The main SARs that mediate TLR-induced autophagy include p62, NBR187, OPTN, and NDP52, and TLR-induced autophagy eliminates invading pathogens in a ubiquitin-dependent manner [68]. The SARs recognize and bind to the ubiquitinated pathogens; the pathogens are then attached to the PG and subsequently encapsulated in the autophagosome for degradation [69]. Intracellular endosomal TLRs including TLR3, TLR7, TLR8, and TLR9, as PRRs, initiate downstream signaling pathways by detecting the unique double-stranded (ds) RNA, single-stranded (ss) RNA, or dsDNA of invading viruses located in the endosome when the viral particles are endocytosed and the capsid is decapsulated in lysosomes [61]. Notably, in the response of plasmacytoid dendritic cells (pDCs) to certain ssRNA viruses, autophagy contributes to viral nucleic acid detection and type I IFN production via transporting the replication intermediates of these viruses from the cytoplasm to the endosome in which TLR7 resides [70]. The absence of Atg5 in pDCs caused inhibition of the sensing of the vesicular stomatitis virus by TLR7 [70].

Autophagy is not only an important part of innate immunity but also plays a crucial role in viral antigen presentation, impacting adaptive immunity during viral infections [71]. Autophagy participates in the antigen-presenting process of dendritic cells and B cells by regulating the antigen presentation of major histocompatibility complex class II (MHC-II) molecules [72]. Exogenous antigenic peptides, which are derived from lysosome degradation and presented by MHC-II molecules, are transferred to the cell surface to induce a CD4^+^ T-cell response. Autophagy activation enhances the uptake of the peptides derived from pathogens into MHC-II molecules [73]. Deletion of Atg5 disturbs B-cell antigen receptor (BCR) clustering and polarization after stimulation [74]. ATG5 is responsible for the relocalization of the internalized BCR into MHC-II molecules containing compartments and lysosomes into the immunological synapse [74]. The absence of ATG5 caused a decrease in antigen presentation to cognate T cells [74]. Epstein–Barr virus (EBV) nuclear antigen 1 (EBNA1) is the major latent antigen of EBV in lymphoma cells, which is presented by MHC-II molecules and detected by CD4^+^ T cells. Inhibition of autophagy decreased the efficiency of EBNA1 recognition by CD4^+^ T cells [75]. In addition, several lines of evidence indicate that autophagy plays a pivotal role in inflammatory responses and secretion of inflammatory cytokines [69].

### Autophagy with CoV interference

**Fig. 4:**
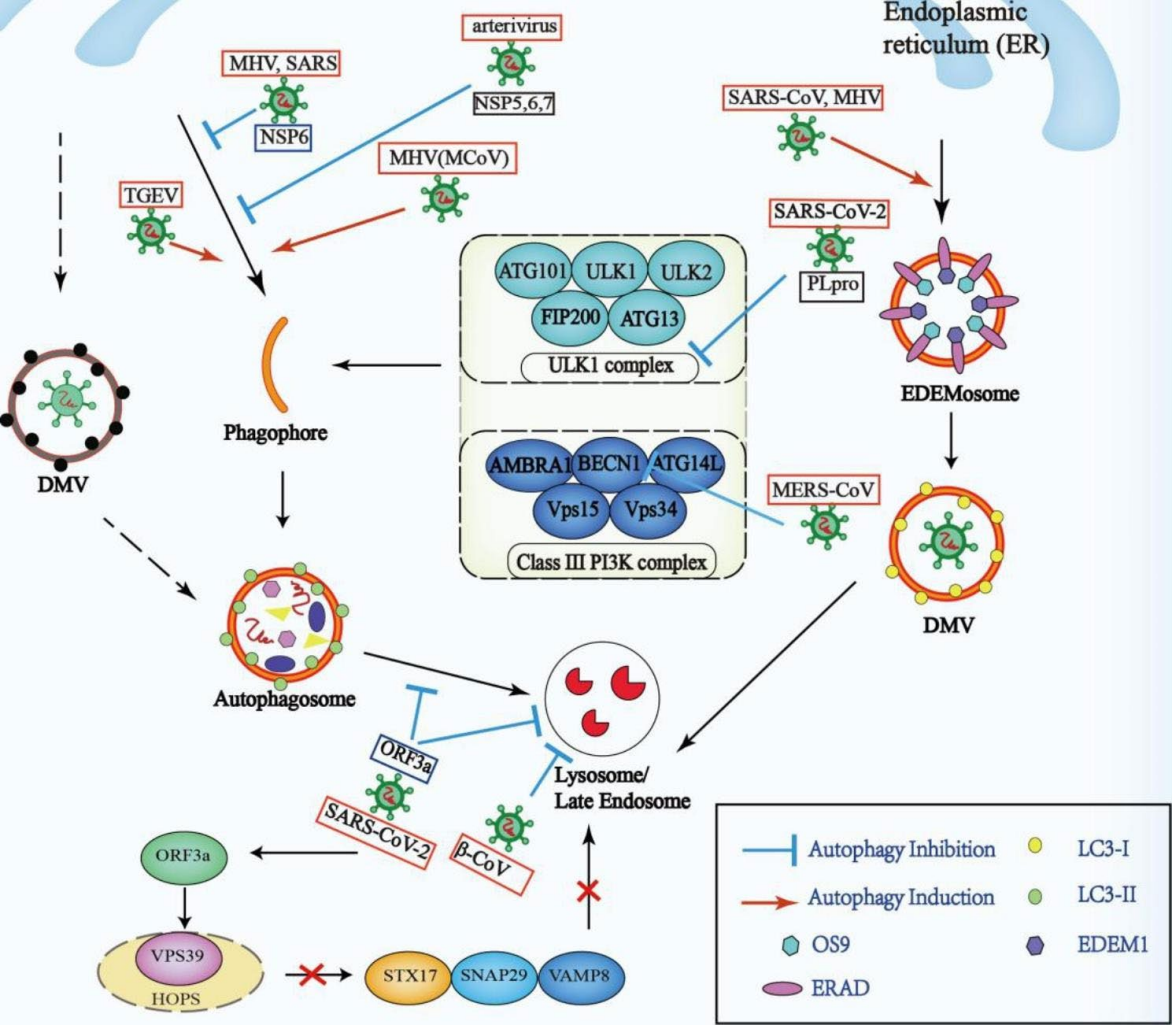
Modulation of coronavirus in the autophagy pathway Although viral infections can trigger autophagy in antiviral immune responses to limit the virus replication cycle and infection process, the surviving viruses can evolve various strategies to inhibit multiple steps of the autophagy pathways or utilize autophagy to escape immune clearance, and even hijack autophagy to provide shelter to their offspring and obtain energy for replication. TGEV infection can induce autophagy. Some studies suggest that pharmacological or genetic inhibition of autophagy increased TGEV replication, whereas other studies have reported that TGEV-activated mitophagy supports cell survival and possibly viral infection. The PL^pro^ of SARS-CoV-2 disrupts autophagy and induces viral pathogenesis by significantly decreasing ULK1 expression. MERS-CoV blocked the autophagic flux by decreasing BECN1 levels mediated by SKP2. IBV or NSP6 protein of MHV and SARS, or NSP5, NSP6, and NSP7 of arterivirus PRRSV could reduce the number of viral components transported from autophagosomes to lysosomes by promoting the generation of autophagosomes with a smaller diameter. The accessory protein ORF3a of SARS-CoV-2 weakens autophagy activity by inhibiting the fusion of autophagosomes/amphisomes with lysosomes. The key to this fusion is the STX17-SNAP29-VAMP8 SNARE complex, whose assembly can be blocked by the interaction of the HOPS complex with ORF3a instead of the autophagosomal SNARE protein STX17. Expression of both SARS-CoV-2 ORF3a and β-coronaviruses can damage lysosomes by impairing their functions including degradation and digestion. The replication of SARS-CoV and MHV could be independent of autophagy, during which the virus hijacked the pathway of EDEMosome formation to produce nonlipidated LC3-I coated DMVs for replication Abbreviations: BECN1: beclin1; DMV: double-membrane vesicle; MCoV: Mouse coronavirus; MERS-CoV: Middle East respiratory syndrome coronavirus; MHV: Mouse hepatitis virus; NSP: nonstructural proteins; ORFs: open reading frames; PL^pro^: papain-like protease; SARS-CoV-2: severe acute respiratory syndrome coronavirus 2; SKP2: S-phase kinase-associated protein 2; TGEV: transmissible gastroenteritis virus; ULK1: unc-51 like autophagy activating kinase 1

Although autophagy can limit viral infections, the surviving viruses have evolved various strategies to inhibit multiple steps of the autophagic pathways or utilize autophagy to escape immune clearance. Furthermore, viruses can hijack the autophagic process to provide shelter to their offspring and obtain energy for replication (Fig. 4).

In a study of infection caused by transmissible gastroenteritis virus (TGEV), an α-CoV, which induced autophagy, the pharmacological or genetic inhibition of autophagy further increased TGEV replication, indicting a negative relationship between autophagy and TGEV replication [76]. However, another study reported a positive relationship, wherein TGEV infection could activate mitophagy to support cell survival and possibly viral infection [77].

The invasion of MERS-CoV blocked the autophagic flux by decreasing BECN1 levels. The proteasomal degradation of BECN1 could be mediated by S-phase kinase-associated protein 2 (SKP2), a type of E3 ligase inducing BECN1 ubiquitination, whereas the inhibition of SKP2 was shown to enhance autophagy and reduce the replication of MERS-CoV [78]. When cells were infected by IBV or NSP6 proteins of mouse hepatitis virus (MHV) and SARS or NSP5, NSP6, and NSP7 of the arterivirus PRRSV, the number of viral components transported from autophagosomes to lysosomes reduced. This result was due to the smaller diameter of autophagosomes induced from the ER, although the number of autophagosomes increased in this process [79].

A study using MHV-A59 as a β-CoV model showed that the canonical autophagy-regulating kinase ULK1 had a dual role; it activated pro-viral functions during early replication but inactivated these functions at the late stages. Further research identified ULK1 as a novel bonafide substrate of SARS-CoV-2 PL^pro^, which significantly decreases ULK1 expression, disrupts autophagy, and induces viral pathogenesis [80].

Another study reported that the accessory protein ORF3a of SARS-CoV-2 decreases autophagy activity by inhibiting the fusion of autophagosomes/amphisomes with lysosomes. The key to this fusion is the STX17-SNAP29-VAMP8 SNARE complex, whose assembly can be blocked when the HOPS complex interacts with ORF3a instead of the autophagosomal SNARE protein STX17. In addition, the expression of ORF3a damages lysosomes, thereby impairing their functions including degradation and digestion [81]. Unlike SARS-CoV-2, the ORF3a of SARS-CoV could not interact with HOPS or inhibit autolysosome formation, suggesting that the autophagy regulation mechanisms are different in SARS-CoV-2 and other types of CoV [81]. Furthermore, it was observed that β-CoV could exploit endosomes/lysosomes for egress, leading to deacidification of lysosomes, inactivation of degradation enzymes, and disruption of antigen-presentation pathways [82]. Moreover, a recent study demonstrated that SARS-CoV-2 infection-induced accumulation of autolysosome facilitates progeny virus propagation, whereby the ORF7a protein initiates autophagy via the AKT-MTOR-ULK1-mediated pathway, but the completion of autophagy is impaired due to decreased SNAP29 protein expression. In this process, the SNAP29 protein, cleaved at aspartic acid residue 30 via caspase 3 activated by ORF7a, blocks the fusion of the autophagosome with lysosomes to promote viral replication [83].

By analyzing the cytoplasmic tails of ACE2 and integrins, both of which were suggested as SARS-CoV-2 receptors [84, 85], many short linear motifs were identified, which were believed to have potential roles in endocytosis, autophagy, and cell signaling [86]. Furthermore, both integrin β3 and ACE2 have LIR motifs associated with selective autophagy [86], and these proteins can directly recruit autophagy cargo [87]. These studies provide potential links between SARS-CoV-2 receptors and endocytosis and autophagy in viral entry and propagation.

MHV is also known as mouse coronavirus (MCoV). A recent study showed that both Atg5 and intact autophagy are not necessary for MHV replication or release in bone marrow-derived macrophages (BMMphi) [88]. This study challenged some early work using MHV, which revealed that infected MHV induces the development of DMVs similar to autophagosomes for their RNA replication, wherein Atg5 plays a central role [89, 90]. The understanding of the source of DMVs induced by MHV can explain this phenomenon, which originates from the ER. During infection, MHV hijacks the pathway of EDEMosome formation to produce nonlipidated LC3-I coated DMVs for replication [91].

Presently, through pharmacological interventions of the ERK/MAPK and PI3K/AKT/mTOR pathways, both of which are linked to autophagy, researchers have found that the inhibition of these pathways decreased MERS-CoV replication [92]. The other effects of autophagy modulators on CoV are summarized in Table 2. Intriguingly, intermittent fasting (IF) was proposed as a promising preventive approach against COVID-19 because it triggers immunomodulatory potential by promoting autophagy [14]. It should be noted that different viruses, diverse cells tested, and even the various techniques used in the studies of autophagy can lead to discrepancies in the result. Although whether and how autophagy contributes to the infection of CoVs remain unclear, manipulating autophagy may be a promising therapeutic strategy for intracellular elimination of pathogens.

**Table 2:** Effect of autophagy modulators on coronavirus

Coronavirus	Cell lines	Protein/pathway	Main results	Refs
MHV	DBT cells	ATG5	Autophagy is required for coronaviruses replication complexes.	[125]
HCV	C5B cells	ATG5	Autophagy is required for HCV replication.	[126]
MHV	MEFs	ATG5	ATG5 is not required for MHV‑A59 replication.	[88]
MHV	HeLa, MEFs	LC3-I	LC3-I rather than intact autophagy process is required for MHV replication.	[91]
PEDV	HEK293T cells	MARCHF8, ATG5	Inhibition of selective autophagy pathway promotes PEDV replication.	[127]
MERS-CoV	HuH7 cells	ERK/MAPK, PI3K/AKT/mTOR	Inhibition of ERK/MAPK and PI3K/AKT/mTOR pathways inhibits MERS-CoV replication.	[92]
HCoV-NL63	HEK 293T, HeLa and MCF-7 cells	LC3, BECN1	PLP2-TM triggers incomplete autophagy process by interacting with BECN1 thereby modulating virus replication and antiviral innate immunity.	[128]
PEDV	IPEC-J2 cells	PI3K/AKT/mTOR	Autophagy induced by NSP6 of PEDV promotes virus replication via PI3K/AKT/mTOR pathway.	[129]
MERS-CoV	HEK293T cells	BECN1	MERS-CoV invasion reduces BECN1 levels and autolysosome formulated for virus replication.	[78]
MHV-A59 and SARS-CoV-2	Murine 17Cl1 fibroblasts	ULK1	PL^pro^ of MHV-A59 and SARS-CoV-2 cleaves ULK1 and thus disrupting autophagy and inducing viral pathogenesis.	[80]
SARS-CoV-2	HeLa, Vero-E6 and HEK293T cells	UVRAG	SARS-CoV-2 ORF3a induces an incomplete autophagy response by interacting with UVRAG.	[130]

**Abbreviations**: ATG: autophagy-related; BECN1: beclin1; CoV: coronavirus; DBT: delayed brain tumor; HCV: Hepatitis C virus; MEFs: mouse embryonic fibroblasts; MERS: Middle East respiratory syndrome; MHV: Mouse hepatitis virus; mTOR: mechanistic target of rapamycin kinase; NSP: nonstructural protein; ORF: open reading frame; PEDV: Porcine epidemic diarrhea virus; PI3K: class I phosphoinositide 3-kinase; PL^pro^: papain-like protease; PLP2-TM: membrane-associated papain-like protease 2; SARS-CoV-2: severe acute respiratory syndrome coronavirus 2; ULK1: unc-51 like

## Autophagy as a potential target for treating COVID-19

Because of the recent COVID-19 pandemic, the role of autophagy in CoV infection has garnered increasing attention, and the autophagic pathways have emerged as a potential target for the development of antiviral drugs against SARS-CoV-2 (Table 3).

**Table 3:** Registered clinical trials to investigate the role of autophagy for COVID-19.

Drugs	Disease conditions	mechanism	Interventions	Status	Phase	NCT number
GNS651	COVID-19 Advanced or Metastatic Hematological or Solid Tumor	Inhibit late-stage autophagy.	GNS651 Standard of care Avdoralimab Monalizumab	Completed	Phase II	NCT04333914
Nitazoxanide	COVID-19	Inhibit late-stage autophagy.	Nitazoxanide Placebo	Terminated	Phase II Phase III	NCT04523090
HCQ	COVID-19	Interfere with autophagosome-lysosome fusion.	HCQ & Arm A vs. HCQ&Arm B	Withdrawn	Not Applicable	NCT04374903
P2Et	COVID COVID-19	Induct complete autophagy.	P2Et Placebo	Recruiting	Phase II Phase III	NCT04410510
Chloroquine phosphate	COVID-19	Interfere with autophagosome-lysosome fusion.	Chloroquine phosphate	Unknown	Phase I	NCT04443270
Chloroquine Hydroxychloroquine	COVID-19 Coronavirus	Interfere with autophagosome-lysosome fusion.	Chloroquine Hydroxychloroquine Standard care	Completed	Phase III	NCT04420247
Chloroquine Phosphate Tablets	COVID-19 Pneumonia	Interfere with autophagosome-lysosome fusion.	Chloroquine Phosphate Tablets Losartan	Withdrawn	Phase II	NCT04428268
Chloroquine Sulfate Hydroxychloroquine	COVID-19	Interfere with autophagosome-lysosome fusion.	Chloroquine Sulfate Hydroxychloroquine Standard supportive care	Terminated	Phase IV	NCT04362332
Rapamycin	Acute Lung Injury/ARDS Respiratory Failure COVID-19	Activate autophagy via inhibiting mTOR.	Rapamycin Placebo	Withdrawn	Phase I Phase II	NCT04482712

**Abbreviations**: ARDS: Acute Respiratory Distress Syndrome; COVID-19: coronavirus disease 2019; HCQ: Hydroxychloroquine; P2Et: Caesalpinia spinosa extract

### Effect of CQ/HCQ on viral infections

Chloroquine (CQ) and its less toxic derivative hydroxychloroquine (HCQ) are FDA-approved drugs that are widely used for the treatment and prevention of malaria. These drugs are also used for the treatment of rheumatoid arthritis and lupus erythematosus. They exhibit potential broad-spectrum antiviral activities against various RNA and DNA viruses [93–95]. These drugs interfere with lysosomal activity and autophagy by decreasing autophagosome–lysosome fusion, which is the main mode of their action [96, 97]. CQ and HCQ are presently considered potential therapeutic agents for COVID-19. In vitro studies have confirmed the inhibitory effects of CQ and HCQ on SARS-CoV-2 infection [98, 99]. Presently, many clinical trials with CQ or HCQ have been initiated. CQ phosphate showed significant efficacy and acceptable safety against pneumonia induced by COVID-19 in multicenter clinical trials performed in China [93]. Patients with moderate COVID-19 treated with HCQ (400 mg/day for 5 days) in combination with conventional treatments showed an improvement compared with the control group with conventional treatments [100]. A retrospective study with 550 critically ill COVID-19 patients revealed that HCQ in combination with the basic treatments, including antiviral drugs and antibiotics, can decrease fatality of patients by attenuating the inflammatory cytokine storm [101]. However, because of cardiac concerns and other serious side effects of CQ or HCQ, FDA has warned that CQ or HCQ should not be used for the treatment of COVID-19 except for hospitalized patients or clinical trial volunteers. Many clinical studies have shown that using HCQ alone or with other agents could not improve the clinical status or decrease the mortality of patients with COVID-19 [48, 102]. Moreover, HCQ cannot prevent symptoms compatible with COVID-19 or be used as postexposure prophylaxis for individuals exposed to confirmed COVID-19 cases [44, 103]. Based on previous preclinical and clinical studies, Edelstein et al. warned that CQ or HCQ should not be used in patients with SARS-CoV-2 infection and acute organ injury (including AKI) [45].

### Effect of other autophagy modulators on COVID-19

Apart from CQ or HCQ, other autophagy inhibitors have been studied to investigate the effect of targeted autophagy on patients with COVID-19. MK-2206, an AKT inhibitor, can activate autophagy via the AKT/mTOR pathway [46]. Inhibition of AKT/mTOR by MK-2206 was reported to significantly reduced virus production [46]. Ivermectin, an FDA-approved anti-parasitic agent, has been reported to induce autophagy by blocking the PAK1/ATK axis or AKT/mTOR signaling pathway and possesses a broad antiviral activity [47, 49, 50]. Ivermectin is effective in inhibiting the replication of SARS-CoV-2 in vitro [50]. GNS561, an inhibitor of autophagy, has been found to possess an antiviral effect against two SARS-CoV-2 strains in vitro and the combination of GNS561 with remdesivir showed a strong synergistic antiviral activity against SARS-CoV-2 [104]. On the contrary, azathioprine, an mTOR inhibitor used for the treatment of inflammatory bowel disease, can prolong clinical illness, delay virus clearance, and decrease serum neutralization antibody titers of SARS-CoV-2 in ferrets [51, 105]. In an ex vivo human lung tissue culture model, class III PI3-kinase inhibitor VPS34‐IN1 and its bioavailable analog VVPS34‐IN1, both of which are autophagy inhibitors, inhibited SARS‐CoV‐2 infection [106].

As autophagy plays a complex role in modulating innate and adaptative immune responses and in the process of virus replication and escape, the outcomes of inhibition or activation of autophagy in treating COVID-19 can depend on various factors. Further in vitro and in vivo studies and clinical trials are required to establish the therapeutic value and benefits of targeting autophagy. Recently, the effects of autophagy regulators other than CQ and HCQ on COVID-19 were determined in vitro [107]. Exogenous administration of autophagy-targeted compounds, including the BECN1-stabilizing anthelmintic drug niclosamide, the selective AKT1 inhibitor MK-2206, and the polyamines spermidine and spermine, can inhibit SARS-CoV-2 propagation in vitro, which shows great potential for the treatment of COVID-19 [107].

## Outlook and challenges

SARS-CoV-2 has been spreading for more than three years. This has seriously affected public health and the global economy. Even though many vaccines against SARS-CoV-2 have been approved, the fast-spreading variants of SARS-CoV-2 can reduce the efficacy of the developed antibodies and vaccines [108–110]. Therefore, SARS-CoV-2 biology and the mechanisms underlying virus–host cell interactions should be studied to find potential interventions for patients with COVID-19. Autophagy, a conserved mechanism for homeostasis maintenance in eukaryotic cells, is widely involved in cell growth, development, immunity, infection, and other physiological processes. During the last decade, studies have revealed the pivotal role of autophagy in antiviral immune responses. Autophagy can control viral infections at multiple steps by degrading intracellular viruses and facilitating innate pathogen detection, inflammatory responses, and antigen presentation. Interestingly, viruses have developed mechanisms to inhibit or hijack autophagy to survive.

Presently, the molecular mechanism of autophagy and its relationship with SARS-CoV-2 infection have been investigated [111, 112]. The therapeutic potential of many agents targeting autophagy for the treatment of COVID-19 has been reported. Among them, CQ and HCQ, which inhibit lysosomal activity and autophagic pathways, have therapeutic potential for the treatment of COVID-19 and are approved by the FDA. However, evidence suggests that SARS-CoV-2 can also impede autophagy activity by blocking the fusion of autophagosomes with lysosomes [81]. Therefore, the activation of autophagy can inhibit SARS-CoV-2 replication and propagation [113, 114]. Exploiting the biological activity-based modeling (BABM) approach and a cell culture live virus assay, researchers have identified many compounds with potential activity against SARS-CoV-2. Surprisingly, most of the confirmed anti-SARS-CoV-2 compounds could inhibit viral entry inhibitors and/or modulate autophagy [115].

To summarize, the development of new drugs and vaccines against COVID-19 may require considerable time; thus, repurposing the existing drugs or related therapeutic target seems to be the best strategy to develop effective therapeutics against COVID-19. This review aims to trigger the interest in the field of autophagy as an antiviral target against viral pathogens (with an emphasis on COVID-19). We summarized the classification and structure of coronaviruses and the process of SARS-CoV-2 infection and replication; provided the common understanding of autophagy; reviewed interactions between the mechanisms of viral entry/replication and the autophagy pathways; and discussed the current state of clinical trials of autophagy-modifying drugs in the treatment of SARS-CoV-2 infection to support our hypothesis. We anticipate that this review will contribute to the rapid development of therapeutics and vaccines against COVID-19. However, the decision to inhibit or activate autophagy for treating COVID-19 should be made by considering different factors. Further studies are warranted to explore the molecular mechanisms of SARS-CoV-2–host–autophagy interplay and investigate whether autophagy inducers/inhibitors exert anti-SARS-CoV-2 effects. Studying the molecular regulation of autophagy during SARS-CoV-2 infection is crucial for developing potential strategies to combat the COVID-19 pandemic.

## Data Availability

Not applicable.

## Abbreviations

ACE2: Angiotensin-converting enzyme 2
ARDS: Acute respiratory distress syndrome
ATG: Autophagy-related
AVs: Autophagic vesicles
BCR: B-cell antigen receptor
BECN1: Beclin 1
CMA: Chaperone-mediated autophagy
COVID-19: Coronavirus disease 2019
CoVs: Coronaviruses
CQ: Chloroquine
DMVs: Double-membrane vesicles
E: Envelope
EBV: Epstein–Barr virus
ER: Endoplasmic reticulum
ERGIC: ER–Golgi intermediate complex
FDA: Food and Drug Administration
gRNA: Genomic RNA
HCQ: Hydroxychloroquine
LC3-II: LC3 lipid
LIR: LC3/GABARAP-interacting region
M: Membrane
MCoV: Mouse coronavirus
MERS-CoV: Middle East respiratory syndrome coronavirus
MHC-II: Major histocompatibility complex class II
MHV: Mouse hepatitis virus
MyD88: Myeloid differentiation primary response gene 88
N: Nucleocapsid
NSP: Nonstructural proteins
ORFs: Open reading frames
PAMPs: Pathogen-associated molecular patterns
PAS: Phagophore assembly site
pDCs: Plasmacytoid dendritic cells
PG: Phagophore
PRRs: Pattern recognition receptors
Ptdins3P: Phosphatidylinositol 3-phosphate
RdRP: RNA-dependent RNA polymerase
S: Spike glycoprotein
SARs: Selective autophagy receptors
SARS-CoV-2: Severe acute respiratory syndrome coronavirus 2
sgRNA: Subgenomic RNA
SKP2: S-phase kinase-associated protein 2
SQSTM1: Sequestosome 1
TGEV: Transmissible gastroenteritis virus
TLRs: Toll-like receptors
TMPRSS2: Transmembrane protease serine 2
TRIF: TIR domain-containing adaptor molecule 1
UIM: Ubiquitin-interacting motif
WHO: World Health Organization
